# Histomorphometry and sperm quality in male rats exposed to 2.45 GHz Wi-Fi

**DOI:** 10.1530/REP-25-0048

**Published:** 2025-04-18

**Authors:** Sivasatyan Vijay, Siti Fatimah Ibrahim, Khairul Osman, Aini Farzana Zulkefli, Mohd Farisyam Mat Ros, Norazurashima Jamaludin, Syed Muhamad Asyraf Syed Taha, Atikah Hairulazam, Farah Hanan Fathihah Jaffar

**Affiliations:** ^1^ Department of Physiology, Faculty of Medicine, Universiti Kebangsaan Malaysia (UKM), Cheras, Federal Territory of Kuala Lumpur, Malaysia; ^2^ Forensic Science Program, Center for Diagnostic, Therapeutic, & Investigative Studies (CODTIS), Faculty of Health Sciences, Universiti Kebangsaan Malaysia (UKM), Federal Territory of Kuala Lumpur, Malaysia

**Keywords:** RF-EMF, Wi-Fi, sperm quality, testes, seminal vesicle, epididymis

## Abstract

**In brief:**

Numerous studies have examined the impact of 2.45 GHz Wi-Fi exposure on the testes, highlighting concerns about its potential effects on male fertility. This study extends the investigation beyond the testes to include the epididymis, seminal vesicle and coagulating gland, providing a more comprehensive understanding of how 2.45 GHz Wi-Fi influences the male reproductive system.

**Abstract:**

Numerous studies have documented the effect of 2.45 GHz Wi-Fi exposure on the testes and sperm quality. Nevertheless, detailed histological alterations of other male reproductive organs are underexplored. Therefore, this study aimed to evaluate detailed histological alterations of the testes, epididymis, seminal vesicle, coagulating organ and sperm parameters following 2.45 GHz Wi-Fi exposure. Eighteen adult male Sprague Dawley rats (*n* = 18) were equally divided into three groups (*n* = 6): control, 4 h and 24 h groups. The groups were exposed to an active router daily for 4 or 24 h, respectively. The control group was sham-exposed using an inactive router. The exposure lasted for 8 weeks at a 20 cm distance, with a power density of 0.141 W/m^2^ and a specific absorption rate of 0.41 W/Kg. Histological findings revealed vacuolation in the testes and the corpus epididymis of the 4 and 24 h groups. The seminal vesicle in both exposed groups exhibited multifocal atypical hyperplasia. Besides, the seminiferous tubule diameter decreased gradually in both exposed groups, with a substantial decrease in the 24 h group. The spermatogenesis index in 4 and 24 h groups also reduced significantly. The latter result was reflected in the sperm concentration, where both groups showed a significant reduction compared to the control group. Sperm motility also decreased significantly in the 4 h groups. Interestingly, there was a substantial increase in sperm viability in the 24 h group. These findings indicate that 2.45 GHz Wi-Fi exposure causes changes in the histology and histomorphometry measurement and impairs important sperm parameters. This highlights the consequences following Wi-Fi exposure on male reproductive health.

## Introduction

Wireless communication has become an indispensable part of daily life. Wireless fidelity, commonly known as Wi-Fi, is a significant technological breakthrough that provides unparallelled convenience and allows this communication. In today’s modern world, Wi-Fi has become a tool that allows users to socialise with friends, participate in online courses and work meetings, stream movies and indulge in online shopping. Based on its wide application, a significant portion of users, approximately 26.6%, dedicate at least 1 to 4 hours daily to internet browsing. In comparison, 11.7% spend more than 18 h online (Malaysian Communications and Multimedia Commission 2020) (http://www.mcmc.gov.my). Thus, the significant dependence on this technology is highlighted.

Devices will communicate wirelessly through radio waves. The most commonly applied radio waves in today’s technology are 2.45 GHz frequency. The transmission of energy through radio waves with a range that varies from 3 kHz to 300 GHz is known as radiofrequency electromagnetic radiation (RF-EMR). Despite being classified as non-ionising radiation, RF-EMR emitted by Wi-Fi and wireless devices still has the potential to be absorbed by body tissues (Yadav *et al.* 2021). This is due to the properties of the 2.45 GHz frequency that operates at microwave wavelengths and can penetrate deep into body tissues, reaching over 50 mm into the human brain (Liu *et al.* 2022, Marar *et al.* 2024). Since the male gonads are only protected by a thin layer of scrotal skin, RF-EMR may penetrate even deeper into the tissue. The absorption of this energy may potentially cause detrimental changes to the spermatogenesis and affect sperm quality.

Therefore, excessive Wi-Fi use nowadays raises health concerns as the testes are one of the most sensitive organs to the RF-EMR aside from the brain and skin (Kesari *et al.* 2018, Narayanan *et al.* 2019, Kim *et al.* 2021). Numerous studies have reported the impact of 2.45 GHz Wi-Fi radiation on the testes, uncovering a range of complex alterations. These alterations include structural and histological changes such as desquamation of germ cells, reduced diameter and occurrence of irregular shape seminiferous tubules and decreased germinal epithelium height (Jonwal *et al.* 2018, Almášiová *et al.* 2021, Andrašková *et al.* 2022). Moreover, various reports have explored the effects of RF-EMR emitted by Wi-Fi devices on sperm quality, reproductive hormones and oxidative stress status (Avendaño *et al.* 2012, Kesari *et al.* 2018, Shahin *et al.* 2018, Jaffar *et al.* 2022, Chu *et al.* 2023).

Assessing testicular alterations and sperm quality is essential when addressing male reproductive health. However, detailed evaluation of other male reproductive organs, such as the epididymis, seminal vesicle and coagulating gland, is equally imperative. Despite its importance, scientific reports regarding these organs remain limited. Sparse studies have addressed the intricate dynamics of these organs and their impact on male reproductive health following Wi-Fi exposure. One study reported the effect of RF-EMR at a frequency of 1.95 GHz on the testes, seminal vesicles, epididymis and prostate (Imai *et al.* 2011).

Nevertheless, the 2.45 GHz frequency is widely employed in contemporary Wi-Fi and wireless devices, making it a ubiquitous standard. Therefore, this animal model study examines its effect on the male reproductive organs, including the testes, seminal vesicle, epididymis and coagulating gland (anterior prostate), and sperm parameters. A comprehensive evaluation of these effects will help assess potential reproductive health risks and support informed decisions on the regulation and use of wireless technology.

## Materials and methods

### Sample and sample size

A total of 18 (*n* = 18) adult male Sprague Dawley rats of 8-weeks-old with an initial body weight of around 250 ± 50 g were obtained from the Laboratory Animal Resources Unit (LARU), Faculty of Medicine, Universiti Kebangsaan Malaysia (UKM). Each rat was housed individually in a well-ventilated plastic cage (43 cm length × 16 cm wide × 29 cm height) in the animal house of Fakulti Sains dan Teknologi, UKM. The ambient temperature was maintained at 23 ± 5°C with a 12 h light:12 h darkness cycle. Food pellets and tap water were provided *ad libitum*, and the rats were monitored daily for behavioural changes. The UKM Animal Ethical Committee (UKMAEC) approved all animal procedures implemented in this study with the approval reference number FP/2023/FARAH HANAN/15-FEB./1305-FEB.-2023-SEPT.-2024.

### Wi-Fi exposure setting

All the animals were divided randomly and equally into three groups, with six rats (*n* = 6) in each group. The first group was the control group, where the rats in this group were sham-exposed towards an inactive Wi-Fi router. The other two groups were exposed to 2.45 GHz Wi-Fi for 4 and 24 h daily, respectively. All animals were housed in the same room but at different time frames to accommodate this experimental setting.

The animal cages were placed at a 20 cm distance from a Wi-Fi router, and they could move freely inside the cage without any movement restrictions. The router was positioned at the same height as the cages, with only the cage walls and its metal coverings as barriers in between. All animals were maintained under these conditions for 8 consecutive weeks.

The exposure was conducted using a TP-LINK AC750 Wireless Dual Band Router Archer C20 (China). This router has three omnidirectional antennas, two generating 2.45 GHz Wi-Fi frequency and one supporting dual frequency, 2.45 and 5 GHz. Only the antenna emitting 2.45 GHz Wi-Fi frequency was activated while the single 5 GHz antenna was deactivated. According to the maximum permissible exposure report for this router, the power density of the antenna is 0.141 W/m^2^ at a constant antenna gain of 3 dBi. The estimated time-averaged whole-body exposure-specific absorption rate is 0.41 W/kg.

For the exposed groups, the Wi-Fi router was actively communicating with a Raspberry Pi computer (Cambridge University, England) using a ping protocol via the Bitvise SSH client version 8.18 software (Slovenia, Hungary and the USA) through 802.11b/g/n standards.

### Animal euthanisation and tissue sampling

After an 8-week exposure to Wi-Fi, animals were euthanised using ketamine (3.34 mg/kg, USA), xylazine (3.34 mg/kg, USA) and Zoletil-50 (1.66 mg/kg, Virbac, Australia) (KTX) cocktail, which was administered intraperitoneally. The death of the animal was confirmed by loss of righting reflex, cardiac arrest, loss of tail pinch reflex, loss of forelimb and hindlimb pedal withdrawal reflexes, and absence of corneal reflex (Jaffar *et al.* 2021). Both sides of the testes, epididymis, seminal vesicle and coagulating gland were meticulously dissected and cleaned of surrounding adipose tissue before being weighed and measured.

### Gross organ morphology and organ coefficient

The harvested organs were photographed on the scale board before their gross morphology was recorded. Immediately after recording each organ’s gross morphology, the organ was weighed, and the organ coefficient for each dissected reproductive organ was calculated using the following formula (Feng *et al.* 2015):

$$\frac{\text{Wet}\;\text{weight}\;\text{of}\;\text{the}\;\text{organ}\;\left(g\right)}{\text{Body}\;\text{weight}\;\left(g\right)}\;\times \;100$$

Except for the cauda epididymis, all other organs were preserved in 10% buffered neutral formalin (Merck, Germany). The cauda section of each epididymis was isolated to analyse the sperm parameter.

### Histology assessment of the reproductive organ

All the organs preserved in the 10% buffered neutral formalin were processed using a standard protocol and embedded in paraffin wax (Merck, Germany). Tissues then underwent tissue processing. The process involved tissue undergoing dehydration through a series of alcohol solutions to eliminate water. Xylene was employed to render tissues transparent, facilitating the infiltration of the embedding medium. Dehydrated tissues were then embedded in paraffin wax for sectioning. The embedded tissue was sectioned using a microtome to create 5 μm thick tissue sections. Sections were placed on glass slides and heated to eliminate excess water and promote subsequent tissue adhesion to the slides.

The mounted sections were deparaffinised for the haematoxylin and eosin (H&E) staining to remove the embedding medium. Sections were rehydrated through a series of decreasing alcohol concentrations. Rehydrated sections were immersed in haematoxylin, a basic dye that stains nuclei blue. Subsequently, sections were washed in alcohol to remove excess haematoxylin and enhance contrast. Eosin, an acidic dye, was applied to stain sections, imparting a pink colour to the cytoplasm and other structures. Stained sections underwent dehydration and clearing steps similar to the initial processing. A coverslip was added over the stained sections using a mounting medium. Stained slides were observed under the light microscope (Olympus bx53f, Japan), and the image was captured under 20× magnification.

### Morphometrical assessment of the seminiferous tubules

Morphometrical assessment of the seminiferous tubule was done by measuring the germinal epithelium’s thickness and the seminiferous tubule’s diameter. A total of 30 seminiferous tubules (Schneider *et al.* 2012, Kohn *et al.* 2022) were measured using the ImageJ bundled with the 64-bit Java 8 software, an image processing program developed at the National Institutes of Health and the Laboratory for Optical and Computational Instrumentation (LOCI, University of Wisconsin, USA).

**Figure 1:**
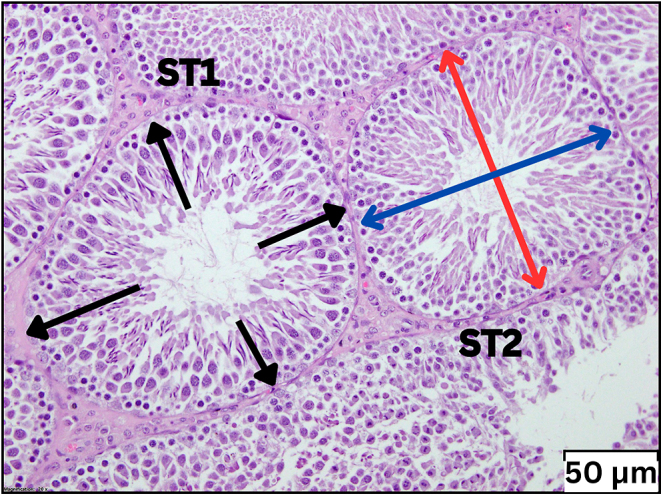
ST1 shows the height of the germinal epithelium in each tubular cross-section, which was measured at four different sites within each tubule to acquire the average measurement. ST2 shows that the seminiferous tubule diameter was measured by selecting the most circular seminiferous tubule by measuring an average of two diameters, D1 (the blue arrow) and D2 (the red arrow), which are perpendicular to each other.

The seminiferous tubule diameter was measured by selecting the most circular-shaped seminiferous tubule in each tissue section. The most circular seminiferous tubule was selected by calculating an average of two diameters, D1 and D2, which were perpendicular to each other. The diameter of each seminiferous tubule was then measured by averaging the readings of D1 and D2, as shown in Fig. 1.

On the other hand, the height of the germinal epithelium in each tubular cross-section was measured at four different sites within each tubule to acquire the average measurement (Saalu *et al.* 2013, Hameed *et al.* 2022). In rodents, the seminiferous epithelium within each seminiferous tubule comprises 14 distinct phases of germ cell development (Griswold 2016). Consequently, the height of the germinal layer varies. Given this fact, the measurement of the germ cell height was standardised by drawing a line from the basement membrane to the head of the spermatid. Specifically, the seminiferous epithelium during phases VIII to XIV was selected as these phases represent key stages of germ cell maturation and spermatid development (Griswold 2016). This approach was consistently applied to all selected seminiferous tubules during measurement.

### Spermatogenesis index

The spermatogenesis index was assessed using the Johnsen score out of 30 seminiferous tubules for each tissue section. Tubular sections were evaluated, each given a score of 1–10 according to the following criteria and index in Table 1. Since this study did not standardize the phase of the seminiferous epithelium cycle during morphometric measurement, assessing the spermatogenesis index was crucial to distinguish the phase in each tubule. This scoring system quantifies spermatogenesis, where higher scores indicate normal function, while lower scores reflect dysfunction (Fu *et al.* 2024). The Johnsen score given was calculated using the following formula (Johnsen 1970, Teixeira *et al.* 2019, Chandni *et al.* 2022):

$$\text{Final}\;\text{Johnsen}\;\text{score},\;\mu =\frac{\sum \left(x\right)\left(y\right)}{z}$$

*x*: a score of each seminiferous tubule; *y*: number of tubules with the assigned score; *z*: total number of evaluated tubules.

**Table 1:** Histological classification of seminiferous tubular cross-sections of Sprague Dawley rats according to the Johnsen scoring system.

Score	Description
10	Complete spermatogenesis with many spermatozoa. Germinal epithelium is organised in a regular thickness, leaving an open lumen
9	Many spermatozoa are present but germinal epithelium is disorganised with marked sloughing or obliteration of the lumen
8	Only a few spermatozoa are present in the section
7	No spermatozoa but many spermatids present
6	No spermatozoa and only a few spermatids are present
5	No spermatozoa, no spermatids but several or many spermatocytes present
4	Only a few spermatocytes and no spermatids or spermatozoa are present
3	Spermatogonia are the only germ cells present
2	No germ cells but Sertoli cells are present
1	No cells in the tubular section

### Sperm collection from the epididymis

The cauda epididymis was isolated and finely minced in 2 mL pre-warmed PBS (Thermo Fisher Scientific, USA). Epididymal sperm were obtained after a 40 min incubation at 37°C, allowing the sperm to swim out of the epididymal tubules.

### Evaluation of sperm parameters

Sperm parameter analysis was done to validate the observed morphological changes in the testicular tissue section.

#### Sperm concentration

A drop of 10 μL sperm suspension was placed on the Makler’s counting chamber (Sefi Medical Instruments Ltd, Israel) and examined under 20× magnification using a bright field microscope (Modified Olympus CH-2, Japan). Sperm concentration was determined using the average of five rows, with each calculation conducted twice.

#### Percentage of motile sperm

A drop of 10 μL sperm suspension was placed on a glass slide and covered with a cover slip. Sperm motility was observed under 20× magnification by using a bright-field microscope (Olympus CH-2, Japan) and categorised into progressive motility (PR), non-progressive motility (NP) and immotility (IM) (World Health Organization 2010). The sperm motility was counted out of 200 sperm in duplicate. The sperm motility was reported as a percentage of motile sperm.

#### Percentage of viable sperm

For sperm viability assessment, the sperm suspension was mixed with a hypo-osmotic swelling test (HOST) solution in a 1:10 ratio. The HOST solution was prepared by adding 0.735 g sodium citrate dehydrate (Sigma Aldrich, Germany) and 1.351 g D-fructose (Sigma Aldrich, Germany) in 100 mL distilled water. This mixture was incubated at 37°C for 30 min, smeared on a microscope slide and dried at room temperature. The smear was stained with Diff-Quick staining to enhance sperm visibility under a bright-field microscope. Viable sperm were counted under 40× magnification, with each counting conducted twice.

### Statistical analysis

The GraphPad Prism 10 (GraphPad Software, USA) (www.graphpad.com) was used for statistical analysis. Parameters such as organ coefficients, morphometric changes in the seminiferous tubules and sperm quality were analysed using one-way analysis of variance (ANOVA), followed by Tukey’s post-hoc test, as these data met the assumptions of normal distribution and homogeneity of variance. For parameters such as the spermatogenesis index, which did not meet these assumptions, the Kruskal–Wallis test was applied as a non-parametric alternative, followed by Dunn’s post-hoc test.

## Results

### Gross morphology and organ coefficient

**Figure 2:**
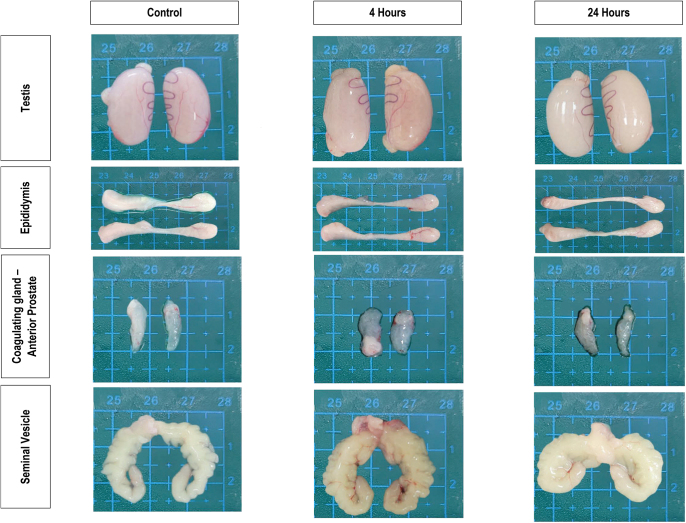
Gross morphology of the male Sprague Dawley’s reproductive organs.

No animal deaths were recorded, and no other signs of behavioural changes were apparent in all groups exposed to 2.45 GHz Wi-Fi for 8 weeks. Furthermore, no gross abnormalities or size differences were recorded in the harvested testes, epididymis, seminal vesicles and coagulating glands (Fig. 2). However, the organ coefficient of the seminal vesicle in the 4 h group showed a significant increase (0.39 ± 0.02, *P* = 0.0147) compared to the control group (0.25 ± 0.03) (Table 2).

**Table 2:** Organ coefficient of the male reproductive organs of Sprague Dawley rats.

Group/organ	Testes	Epididymis	Seminal vesicles	Coagulating gland
Control	0.64 ± 0.05	0.28 ± 0.01	0.25 ± 0.03	0.05 ± 0.00
4 h	0.75 ± 0.02	0.30 ± 0.01	0.39 ± 0.02*	0.05 ± 0.01
24 h	0.63 ± 0.09	0.26 ± 0.03	0.30 ± 0.04	0.05 ± 0.01

The organ coefficient refers to the wet weight of the organ (g)/body weight (g) × 100. Data is presented as the mean ± SEM (*n* = 6).

* Represents a significant difference compared to the control group.

### Histology changes of the testes, epididymis, seminal vesicle and coagulating gland

**Figure 3:**
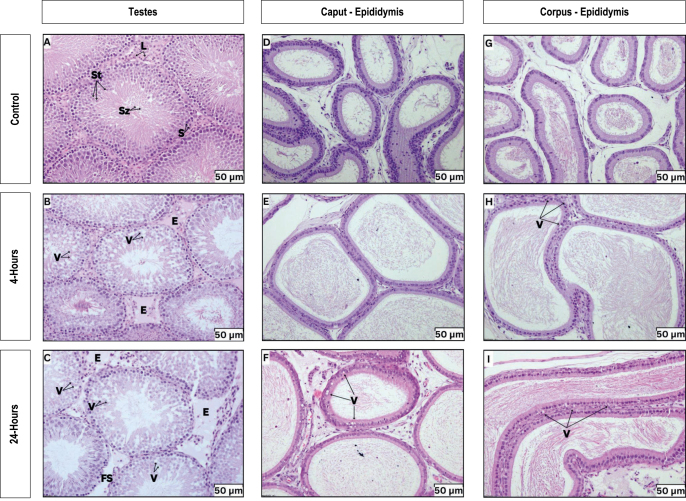
Histopathological findings of the testes and epididymis for each experimental group. (A, B, C) The photomicrograph of the testes cross-section. (D, E, F) The photomicrograph of the cauda and (G, H, I) corpus epididymis cross-section. All figures were recorded under 20× magnification. L, Leydig cells; S, spermatogonia; St, spermatocyte; Sz, spermatozoa; V, vacuolation; E, oedema; FS, free space.

In the histological analysis of the testes in the control group, a typical histological structure of the seminiferous tubules was observed. The tubule and surrounding cells were closely packed together and maintained their integrity. The seminiferous tubules in the control group displayed a regular shape, with Leydig cells in the interstitium. The arrangement of germ cells appeared normal, with spermatogonia residing close to the basement membrane, exhibiting distinct features under staining. Throughout the tubules, various stages of sperm development, from spermatocytes to late spermatids, were appropriately intercalated between Sertoli cells. Spermatozoa were visible within most of the lumens. Only minimal vacuolation was noted (Fig. 3A).

Exposure to 4 h Wi-Fi revealed a tubular arrangement similar to that of the control group, with noticeable vacuolation and interstitial oedema (Fig. 3B). Similarly, the 24 h exposure group displayed significant vacuolation and oedema (Fig. 3C).

In evaluating the epididymis, the caput section showed that the ducts lined with principal pseudostratified columnar cells, closely arranged extending from the basal lamina toward the lumen. These principal cells bear apical stereocilia. Basal cells were interspersed among the principal cells, resting on the basal lamina. These cells were recognized by their oval nuclei surrounded by a scant amount of cytoplasm. A few small, rounded shape with central spherical nuclei with a clear rim of cytoplasm of halo cells also can be found. The ductus epididymis was separated by scanty connective tissue with a moderate amount of sperm present within the lumen (Fig. 3D). The corpus region also exhibited a similar appearance to the caput but with a lower number of columnar epithelial cells and halo cells (Fig. 3G), which is a typical appearance of this region. While the caput epididymis appeared unaffected by Wi-Fi exposure (Fig. 3E), the corpus epithelium exhibited increased vacuolation following both 4 h (Fig. 3H) and 24 h exposures (Fig. 3I).

**Figure 4:**
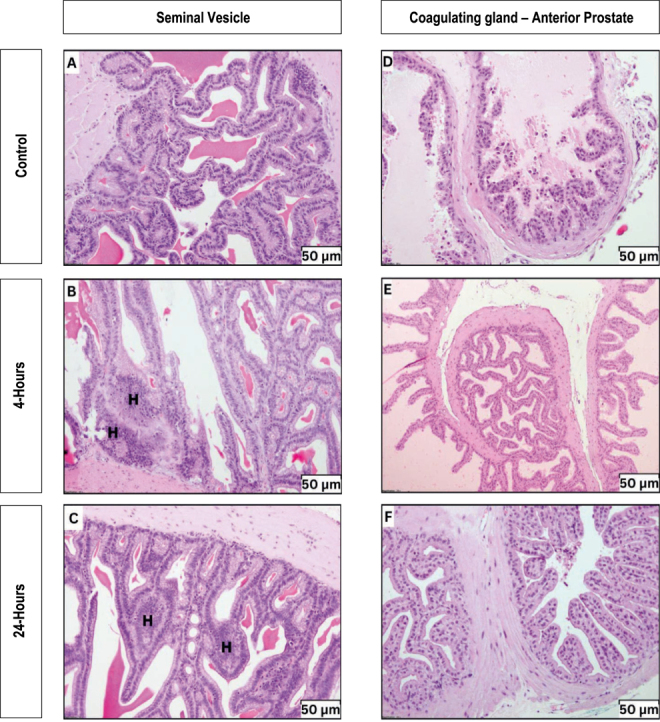
Histopathological findings of seminal vesicle and coagulating gland for each experimental group. (J, K, L) The photomicrograph of the seminal vesicle and (M, N, O) the coagulating gland cross-section. All figures were recorded under 20× magnification. H, hyperplasia; V, vacuolation.

On the other hand, the seminal vesicle of the control group displayed intricate glandular mucosa with a diverse epithelial composition, contributing to secretory material in the lumen (Fig. 4A). Exposure to 4 h Wi-Fi resulted in decreased epithelial cell height. Besides, both exposure durations induced multifocal atypical hyperplasia (Fig. 4B and C), characterised by cuboidal to columnar cells with heightened cytoplasmic basophilia. Conversely, assessment of the coagulating gland revealed no notable differences across experimental groups. All groups demonstrated a consistent epithelial structure and secretory pattern (Fig. 4D, E, F).

### Morphometric changes of the seminiferous tubule

Analysis of the seminiferous tubule diameter demonstrated that 24 h Wi-Fi exposure had a significant decrease (300.70 μm ± 5.17) compared to the control group (323.70 μm ± 2.86, *P* = 0.0129) and 4 h group (318.70 μm ± 3.65, *P* = 0.0159). No significant difference between the control group and the 4 h group was recorded. On the other hand, the thickness of germinal epithelium (μm) in all experimental groups showed no significant differences (Table 3).

**Table 3:** Histomorphometry analysis of the seminiferous tubule of Sprague Dawley rats. Data is presented as the mean ± SEM (*n* = 30).

Group	Thickness of GE (μm)	Diameter of ST (μm)
Control	105.70 ± 1.45	323.70 ± 2.86
4 h	101.70 ± 1.48	318.70 ± 3.65
24 h	97.08 ± 2.21	300.70 ± 5.17*^,^^†^

GE, germinal epithelium; ST, seminiferous tubule.

* Represents a significant difference compared to the control group.

^†^ Represents a significant difference compared to the 4 h group.

### Spermatogenesis index

Evaluation of the spermatogenesis index for each group showed that there was a significant decrease in the spermatogenesis index in the 4 h group (*P* < 0.0001) and 24 h group (*P* < 0.0001) compared to the control group. No significant difference was recorded between the 4 h group compared to the 24 h group (Table 4).

**Table 4:** Spermatogenesis index of the seminiferous tubule of Sprague Dawley rats in each group. Data is presented as the mean ± SEM (*n* = 30).

Group	Spermatogenesis index
Control	7.62 ± 0.12
4 h	6.75 ± 0.12*
24 h	6.23 ± 0.14*

* Represents a significant difference compared to the control group.

### Sperm parameters

**Figure 5:**
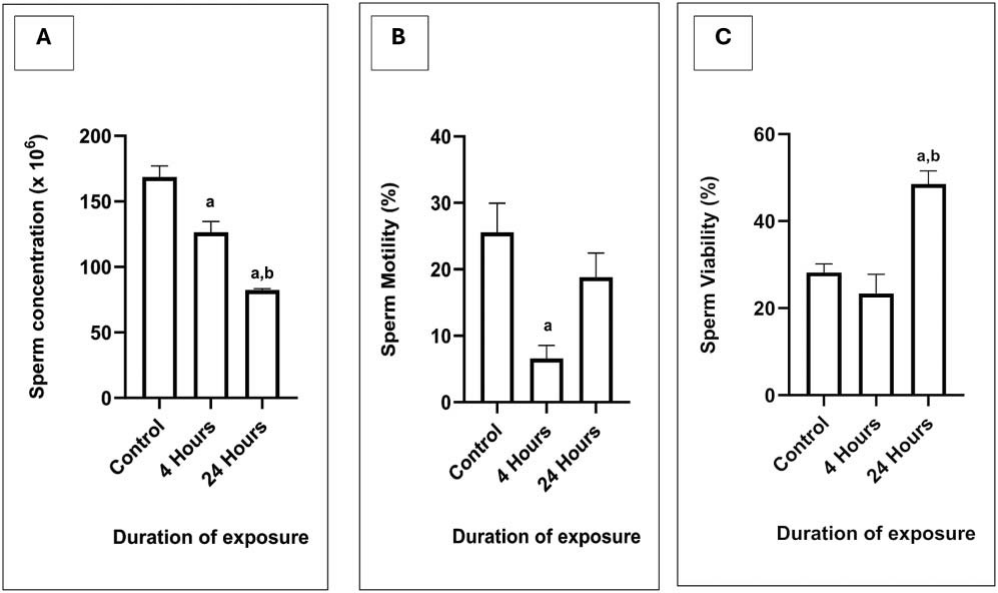
Sperm parameter analysis in each group following exposure. Data are presented as the mean ± SEM, (*n* = 6). (A) Sperm concentration, with the respective duration of Wi-Fi exposure. (B) Percentage of total motile sperm with the respective Wi-Fi exposure. (C) Viable sperm within the respective Wi-Fi experimental exposure groups. ^a^represents a significant difference compared with the control group. ^b^represents a significant difference compared with the 4 h group. ^c^represents a significant difference compared with the 24 h group.

The sperm concentration decreases gradually as the duration of exposure towards the 2.45 GHz Wi-Fi increases. Findings showed that exposure of 4 h (126.5 × 10^6^/mL ± 8.205, *P* = 0.0016) and 24 h (82.25 × 10^6^/mL ± 1.216, *P* < 0.0001) demonstrated a significant decrease in sperm concentration compared to the control group. A significant reduction was also recorded in the 24 h group compared to the 4 h group, *P* = 0.001 (Fig. 5A).

The percentage of sperm motility in the 4 h group (6.583% ± 1.979) showed a significant decrease compared to the control group (25.56% ± 4.399), with a *P*-value of 0.0042. No significant difference was recorded between the control and the 24 h groups (Fig. 5B). The percentage of sperm viability in the 24 h group (48.54 ± 2.987) was statistically increased compared to the control group (28.19% ± 1.969), with *P* = 0.0016, and the 4 h group (23.31% ± 4.473), with *P* = 0.0002, respectively (Fig. 5C).

## Discussion

Despite concerns about male reproductive health, few studies have thoroughly examined the histological changes in male organs due to RF-EMR exposure from wireless devices. Acknowledging this gap, this study evaluated the gross morphology and microscopic changes of male reproductive organs after 2.45 GHz Wi-Fi exposure. Interestingly, no apparent macroscopic abnormalities were recorded. These findings align with previous animal experiments on 1.95 GHz (Imai *et al.* 2011) and 2.45 GHz (Shokri *et al.* 2015) RF-EMR exposure. Both studies reported no significant changes in body weight, macroscopic organ appearance or reproductive organ weights after the exposure.

However, this study recorded a significant increase in the seminal vesicle coefficient in the 4 h exposure group compared to the control group. This finding contradicts the previous report by (Imai *et al.* 2011) who reported no changes in seminal vesicle weight, and (Shokri *et al.* 2015) who observed a significant decrease in its relative weight. Differences in exposure settings, duration of exposure and animal species may account for the discrepancy in outcomes observed between these studies (Kim *et al.* 2014, Jaffar *et al.* 2019).

The experiment was further extended to include a microscopic evaluation of the tissue section. Testicular tissue section in both exposure groups revealed the presence of vacuolisation within the seminiferous tubule and increased interstitial oedema. It was observed that these changes were exacerbated by longer exposure durations of 24 h, suggesting a time-dependent effect. A study by Almášiová *et al.* (2017) reported similar vacuole formation in the seminiferous tubules after rats were exposed to 2.45 GHz RF-EMR. The appearance of vacuoles within the seminiferous tubule indicates early degenerative changes (Creasy *et al.* 2012) and the development of testicular oedema is a common finding in irradiated testes (Porter *et al.* 2006, Yamaguchi *et al.* 2009).

Since RF-EMR is classified as non-ionising radiation, the question arises as to how it causes this effect. The potential mechanism might involve the penetration and absorption of the microwave energy by the testicular tissue, leading to localised tissue heating. This testicular tissue heating could cause a breakdown of the vasculature (Setchell & Breed 2006, Ogundoyin & Oluwatunase 2020), thereby causing vascular leakage that may contribute to the development of interstitial oedema and tubular vacuolation.

Moreover, there were a few evidence that showed the development of oxidative stress following RF-EMR exposure (Jaffar *et al.* 2024, Jamaludin *et al.* 2025). Excessive reactive oxygen species during oxidative stress may cause damaging effects towards the blood–testis barrier cellular membranes (Zeng *et al.* 2025), impairing the tight junction (Mita *et al.* 2011), further contributing to the appearance of oedema and vacuolation in the seminiferous tubule.

Despite 2.45 GHz RF-EMR, even a lower frequency of 900 MHz, which is commonly used by mobile phones, could also cause a notable increase in interstitial oedema and damage to the seminiferous tubules (Nassar *et al.* 2020, Ok *et al.* 2024). Despite these changes, the testes may maintain their standard size and gross appearance during the early stages of degeneration as the degenerative process occurs gradually over time (Yuan & McEntee 1987).

Histomorphometry measurement revealed a significant decrease in seminiferous tubule diameter in the 24 h exposure group, further indicating degenerative signs. Previous studies also reported similar findings, with a considerable reduction in the diameter of the seminiferous tubule following 2.45 GHz RF-EMR exposure (Shahin *et al.* 2014, Delavarifar *et al.* 2020). The observed decrease in the diameter of the seminiferous tubules might be attributed to the apoptosis of germ cells, Sertoli cells and spermatogenic cells (Septiani *et al.* 2022). This apoptosis is likely induced by the combined effect of the thermal changes (Kanter *et al.* 2013) and oxidative stress (Chaki *et al.* 2006) caused by RF-EMR exposure.

Furthermore, the score for the spermatogenesis index was significantly decreased in both exposed groups, further manifesting the degenerative changes of the testicular structure leading to spermatogenesis disturbances. Subsequently, it was reflected in the spermatogenesis outcome, where the present data indicated that when the exposure time increased, the sperm concentration gradually reduced. The decrease in sperm concentration following 2.45 GHz RF-EMR exposure was also widely reported in previous studies (Agarwal *et al.* 2009, Dasdag *et al.* 2015, Kesari *et al.* 2018, Jaffar *et al.* 2019, Maluin *et al.* 2021).

Given that the testes are the most susceptible organs to RF-EMR exposure and a heat-sensitive organ (Mahmoudi *et al.* 2024), the localised tissue heating may impact the high mitotic activity germ cells, as these cells are vulnerable to temperature changes (Sciorio *et al.* 2024). Cumulatively, the histomorphometry changes of the testes and the outcome of the spermatogenesis indicate that exposure to 2.45 GHz Wi-Fi increases the likelihood of male infertility by causing disorganisation in the testicular ultrastructure, even though the testicular gross appearance and weight were unaffected.

Another critical male reproductive structure is the epididymis. This site provides a crucial microenvironment for sperm maturation (Chen *et al.* 2022), where they acquired motility and fertilization capacity (Wu *et al.* 2020). This study’s histological evaluation of the epididymal tissue sections demonstrated that the caput epididymis remained unaffected by exposure. However, the corpus epithelium showed increased vacuolation after 4 and 24 h exposures. This finding contradicts the previous report that used horn antennas for 2.6 GHz exposure (Seymen *et al.* 2021). Earlier reports found vacuoles, disruptions in the normal pseudostratified columnar epithelium lining the tubules, damage to stereocilia and reduced sperm content within the epididymal tubule. The implementation of a horn antenna at 2.6 GHz in the previous report could account for the more severe damaging effects observed on the epididymis compared to our findings. As the epididymis is an essential site for the further development of sperm, alteration of the tubule epithelium may result in infertility.

This study also recorded a significant decrease in sperm motility and viability in the 4 h group. The decline in sperm motility was also a common finding reported by Michael Oni *et al.* (2011), Avendaño *et al.* (2012), Shokri *et al.* (2015) following the RF-EMR exposure. Increasing the exposure time to 24 h was anticipated to worsen both parameters. However, it is interesting to note that both sperm motility and viability increased in the 24 h exposure group. Based on these findings, it was postulated that the epididymis may compensate in certain places while undergoing degenerative changes in another region. In this case, the initial segment of the caput epididymal region, which is a significant player in sperm maturation (Légaré & Sullivan 2020), showed no prominent degenerative changes. Therefore, the caput epididymal region might still perform a proper function to maintain sperm motility and viability, thus contributing to an overall increase in sperm motility and viability recorded in this study. The precise reason for these discoveries would require further investigation into the underlying causes, including a thorough analysis of the molecular structure in epididymal tissue.

Besides the primary male sex organ, histology changes of male accessory glands including the seminal vesicle and the coagulating gland were also evaluated. Both glands produce a secretion that creates a copulatory plug in the female rat during transcervical sperm transport (Nascimento-Gonçalves *et al.* 2022). Previous studies (Imai *et al.* 2011, Dasdag *et al.* 2015, Shokri *et al.* 2015) only reported the weight of each gland but did not evaluate the histological changes. In contrast, this study identified multifocal atypical hyperplasia regions in the seminal vesicles of both exposed groups. The significant increase in the seminal vesicle coefficient observed in the exposed groups may be attributed to these hyperplastic changes. Commonly, seminal vesicle hyperplasia is closely related to the various prostatic lesions (Cimadamore *et al.* 2020, Nascimento-Gonçalves *et al.* 2022). Since no noticeable alterations were discovered on the coagulating gland, often referred to as the anterior prostate, it is unclear how the seminal vesicle hyperplasia in this study relates to it. Additional confirmation of its link may be obtained by assessing the alterations in the other prostatic regions, such as ejaculatory ducts and prostatic urethra.

## Conclusion

This study found that although the macroscopic appearance and organ coefficient of male reproductive organs showed no prominent changes, the microscopic appearance demonstrated degenerative alterations. The degenerative changes were somehow localised in a particular region and exacerbated promptly. The structural changes that occur cause disturbances in spermatogenesis in the testes and sperm maturation in the epididymis. This implies that exposure to 2.45 GHz RF-EMR from wireless communication may harm the reproductive organs due to the deterioration of essential organs’ ultrastructure in the male reproductive system. Underpinning these changes might help pinpoint a precise approach to addressing male infertility associated with current Wi-Fi exposure.

## Declaration of interest

The authors declare that there is no conflict of interest that could be perceived as prejudicing the impartiality of the work reported.

## Funding

This work was funded by Universiti Kebangsaan Malaysiahttps://doi.org/10.13039/501100004515 through Geran Galakan Penyelidik Muda (GGPM) under the grant number GGPM-2022-031.

## Author contribution statement

SV, FJ, SI and KO designed the study. SV, MR, AZ, NJ, ST and AH conducted the data acquisition, interpreted and analysed the data. SV draughted the original manuscript. FJ, SI and KO revised the manuscript and supervised the whole work. All authors read and approved the final manuscript.

## Ethics statement

All procedures involving animals performed in this study were reviewed and approved by the Animal Ethics Committee UKM (UKMAEC) with the approval number: FP/2023/FARAH HANAN/15-FEB./1305-FEB.-2023-SEPT.-2024.
